# Association of Social Vulnerability and Access to Higher Quality Medicare Advantage Plans

**DOI:** 10.1007/s11606-024-09252-1

**Published:** 2024-12-20

**Authors:** Hansoo Ko, Ghaida Alsadah, Gilbert Gimm

**Affiliations:** Department of Health Administration and Policy, College of Public Health, George Mason University, Fairfax, USA

**Keywords:** medicare Advantage, social vulnerability index, high-quality insurance plans, unmet social needs

## Abstract

**Background:**

With more than half of all beneficiaries enrolled in Medicare Advantage (MA) plans, ensuring access to high-quality MA plans is a key concern for policymakers. Access to high-quality MA plans may be limited in certain areas if private insurers are not willing to offer high-quality MA plans in local areas with greater unmet health-related social needs.

**Objective:**

This study examined the association of a market-level social vulnerability index (SVI) score with the number of high-quality MA plans.

**Design:**

This study conducted a retrospective cross-sectional study.

**Participants:**

Our analysis included 3113 USA counties in 2020.

**Main Measures:**

Our primary outcome measure, the availability of high-quality MA plans at the market level, was defined by counting the raw number of 5-star plans, plans with 4.5 or higher stars, and plans with 4 or higher stars. We also counted the number of all MA plans at the market level as an outcome measure to explore private insurers’ market entry and participation decisions.

**Results:**

We found evidence that fewer high-quality MA plans are available in markets with greater unmet social needs (higher SVI scores). Compared to the least vulnerable markets, the most vulnerable markets had 1.5 fewer MA plans overall [95%CI −2.9, −0.1]. The most vulnerable markets also had 1.1 fewer 4 or higher star plans [95%CI −1.9, −0.3] than the least vulnerable markets. Furthermore, this negative association was concentrated in the southern region, which has a greater proportion of Black/African Americans in its market-level populations.

**Conclusion:**

As historically marginalized groups are more likely to reside in markets with greater unmet social needs, disparities in access to high-quality MA plans may widen existing health disparities. Therefore, monitoring the availability of high-quality MA plans in areas with greater unmet social needs is needed to improve health equity for MA beneficiaries.

**Supplementary Information:**

The online version contains supplementary material available at 10.1007/s11606-024-09252-1.

## INTRODUCTION

With more than half of all Medicare beneficiaries enrolled in private Medicare Advantage (MA) plans as of 2023, ensuring access to high-quality MA plans is a key concern for policymakers and the Centers for Medicare and Medicaid Services (CMS). MA enrollment, which has grown rapidly since 2007, is expected to represent more than 60% of all beneficiaries by 2033.^1^

In contrast to traditional Medicare coverage with separate enrollment for Part B and Part D coverage, MA offers a conveniently bundled package designed by private insurers that simplifies enrollment. However, before entering a local market, private insurers must obtain CMS approval that confirms their new MA plan will have an adequate provider network. In addition, the private insurer is required to submit an annual MA plan bid to CMS that estimates the monthly capitated payment needed to cover Medicare Part A and Part B services in the following year. If the bid is higher than the CMS average payment, the private insurer is required by CMS to incorporate the “overbid” in a higher premium relative to competing MA plans. If the bid is lower than the CMS average payment, the private MA plan receives a partial refund (i.e., portion of the difference between the lower bid and average payment) which can be used to support lower premiums.

Some MA plans also have lower premiums and Part D prescription drug benefits that are attractive to Medicare beneficiaries.^2^ Growth in MA enrollment has been particularly strong for Black and Hispanic beneficiaries.^3^ However, a recent study found that low-income and Black beneficiaries are more likely to enroll in MA plans of relatively lower quality on average.^4^

Although MA plans are widely available in most states today, it is not clear if access to *high-quality* MA plans is readily available for Medicare beneficiaries. CMS assesses MA plan quality using a 5-star rating system and posts MA contracts’ star ratings at Medicare.gov.^5^ Each MA contract is rated from 1 to 5 stars on an annual basis based on a weighted average of 46 quality rating scores for measures of administrative performance, including clinical quality, and patient satisfaction. ^6^ Prior studies found differences in patient outcomes and satisfaction measures based on MA plan star ratings such as use of high-quality facilities, readmission rates, and disenrollment rates. ^7–9^

Access to high-quality MA plans may be limited in certain regions of the USA if private insurers are less willing to offer high-quality insurance plans in local areas with greater unmet health-related social needs. Consistent with this hypothesis, prior studies on Affordable Care Act (ACA) Marketplaces found that private insurers’ decisions on market entry and benefit design may exacerbate health disparities. One study found the market-level Black population share was inversely associated with the number of insurance carriers offering private plans.^10^ McManus et al. (2020) found the proportion of qualified Marketplace health plans with restrictive prior authorization requirements for HIV pre-exposure prophylaxis was disproportionately higher in the South.^11^

Under the Quality Bonus Program, CMS has paid high-quality MA plans (rated 4 or higher) a 5% bonus to their monthly capitated payments which can be used to provide extra benefits to MA beneficiaries.^12^ However, this MA payment system may lead some private insurers to avoid operating in regions with a greater share of beneficiaries with complex health conditions or unmet social needs. For example, MA contracts in markets with the highest share of African Americans were found to be less likely to achieve 4 or higher stars. ^13^ Also, private insurers may avoid offering high-quality MA plans in socioeconomically disadvantaged markets where contracting with high-quality providers can be challenging.

Studies have also found evidence of disparities in access to high-quality MA plans. Black and Hispanic Medicare beneficiaries were less likely to enroll in high-quality MA plans.^3^ However, if 5-star plans were available in their communities, Black and Hispanic beneficiaries were more likely to enroll in high-quality plans than their peer beneficiaries.^14^ That said, the association of market-level social vulnerability and the number of high-quality MA plans have not been rigorously studied. As historically marginalized groups tend to reside in markets with greater vulnerability, disparities in access to high-quality MA plans may widen existing health disparities.

To address this gap in knowledge, the current study used market-level MA plan quality ratings to examine the association of a market-level measure of social vulnerability with the count of high-quality MA plans. Specifically, we used the Social Vulnerability Index (SVI), a composite score developed by the Centers for Disease Control and Prevention (CDC) to assess local areas based on their degree of unmet social needs.^15^ CDC constructs SVI at the census tract or county level using 15 variables (socioeconomics, household composition, racial/ethnic minority share of population, and local housing or transportation availability) from the American Community Survey (ACS) estimates. SVI scores have been used to explore associations with cancer screening rates, outcomes for supply-sensitive care, mortality rates, and obesity prevalence.^16–19^

By using a community-level SVI score, we examined if disadvantaged areas have fewer high-quality MA plans. Also, we explored if the association of SVI scores and high-quality MA plans is stronger in certain regions of the USA to reveal evidence on private insurers’ market entry decisions. Finally, results from this study highlight the need for CMS and researchers to monitor the availability of high-quality MA plans by region for the purpose of improving health equity.

## METHODS

### Data

Data for this study were used from two main sources. First, we obtained 2020 market-level SVI data (excluding Puerto Rico) from the CDC public website.^15^ The SVI is a composite index score representing each market’s percentile ranking (range from 0 to 1). We then merged market-level SVI scores with MA plan-by-market-level information on contract/plan identifier, plan types, service areas, and star ratings obtained from CMS Medicare Advantage Landscape File for the contract year 2020.

This study used counties to define a local market. Private insurers contract with CMS to offer MA plans, and each contract can include multiple plans in each local market, which may include a county or region. Our dataset excluded Alaska since 2020 MA plan data were missing for that state. The final analytic sample consisted of 3113 county-level markets with non-missing SVI information.

We counted the total number of MA plans at the market level based on their CMS quality rating (number of stars assigned by CMS). We excluded MA plan types that were only available to subpopulations such as dual-eligible plans, Program of All-Inclusive Care for the Elderly plans, part B-only plans, employer-sponsored plans, or special needs plans. We also excluded MA plans that had missing star rating information.

Our primary outcome measure was defined by counting the raw number of 5-star plans, plans with 4.5 or higher stars, and plans with 4 or higher stars. We also counted the number of all MA plans at the market level as an outcome measure to explore private insurers’ market entry and participation decisions. If private insurers avoid offering any MA plans in highly disadvantaged areas, SVI scores will be negatively associated with the number of MA plans regardless of quality. On the other hand, if MA plans in highly vulnerable markets are less likely to have high star ratings, we will find a negative association of SVI with the number of *high-quality* MA plans, while the association of SVI scores and the total number of MA plans (regardless of star rating) may not be significant. Each of these hypotheses would yield different policy implications.

We used SVI overall composite scores at the market level as our main exposure variable with percentile rankings ranging from 0 (least vulnerability) to 1(highest vulnerability). Following previous studies, we categorized SVI scores into five quintile group categories for our main analysis (very low (SVI scores lower than 0.2), low (0.2 to <0.4), moderate (0.4 to <0.6), high (0.6 to <0.8), very high vulnerability (0.8 or higher)).^16,19^

#### Statistical Analysis

We conducted a descriptive analysis of outcomes and independent variables across markets and by SVI quintile group. We reported unweighted statistics by market population to ensure that more populated markets were not overrepresented. Then we specified multivariate linear regression models to estimate the association of market-level SVI scores and availability of high-quality MA plans. We also performed a subgroup analysis by census region and by rurality status to see if local SVI associations with the availability of high-rated MA plans were stronger in certain regions of the USA. For our subgroup analysis, we grouped markets into SVI quintiles separately for each region.

To account for sociodemographic and healthcare resource factors, which are not included in the SVI measure and might be correlated with geographic variations in insurance markets, we used rurality indicators from the Rural-Urban Commuting Area Codes and health care resource information from the Area Health Resource File. In our analysis, we controlled for rurality status (i.e., rural, micropolitan, or metropolitan), total population, number of active physicians per 100,000 population, number of hospital beds per 1000 population, and the estimated share of households without internet access. We also controlled for state fixed effects to account for region-specific policies that can be associated with market vulnerability and our outcome measures. We used robust standard errors clustered at the state level.

We also conducted sensitivity analyses, first, by running the main regression model with a continuous SVI percentile ranking (0 to 1) measure instead of using quintile SVI measures. This step allowed us to show if the number of high-rated MA plans changes as social vulnerability percentile ranking moves from the least vulnerable (0) to the most vulnerable markets (1). Second, we performed analyses using four subcategories (socioeconomics, household composition, racial/ethnic minority, and housing/transportation factors) of the SVI measure instead of the overall composite score to explore which SVI subcategory has stronger associations with the availability of high-rated MA plans. The overall SVI score was measured with 15 variables from the four themes, and each subcategory has separate market-level vulnerability percentile rankings.

This study was deemed exempt from the Institutional Review Board at George Mason University since it used publicly available market-level datasets. We used a significance level of 5% for our analysis and presented mean values with 95% confidence intervals. We performed statistical analysis in Stata, version 18.

## RESULTS

Table 1 presents market-level descriptive statistics in 2020 by SVI quintile group. Of the 3113 total county-level markets included in our study, 41% were classified as rural, 21% were micropolitan, and the remaining 38% were metropolitan. Compared to markets in the lowest SVI quintile, markets in the highest SVI quintile were more likely to be in the South and West, were more densely populated, and had a larger share of households without Internet access.

**Table 1:** Market-Level Summary Statistics, 2020

	All counties	SVI quintile groups				
		Very low (0 $\le$ SVI $<$ 0.2)	Low (0.2 $\le$ SVI $<$ 0.4)	Moderate (0.4 $\le$ SVI $<$ 0.6)	High (0.6 $\le$ SVI $<$ 0.8)	Very high (0.8 $\le$ SVI $<$ 1)
Number of MA plans						
5-star plans	0.385 [1.242]	0.607 [1.448]	0.486 [1.463]	0.320 [1.084]	0.313 [1.203]	0.197 [0.866]
4.5+ star plans	2.894 [4.376]	3.323 [4.523]	3.562 [5.124]	3.340 [4.828]	2.708 [4.007]	1.532 [2.654]
4+ star plans	8.947 [8.222]	10.097 [8.965]	10.575 [9.242]	9.752 [8.835]	8.358 [7.363]	5.934 [5.114]
All plans	16.290 [10.728]	15.974 [11.704]	17.705 [11.322]	17.633 [11.257]	16.621 [10.421]	13.545 [8.023]
Rurality (1=100%)						
Rural	0.410	0.453	0.406	0.414	0.363	0.413
Micropolitan	0.212	0.159	0.173	0.204	0.241	0.282
Metropolitan	0.378	0.388	0.421	0.382	0.397	0.305
Census region (1=100%)						
Northeast	0.127	0.180	0.191	0.112	0.093	0.057
Midwest	0.316	0.453	0.379	0.350	0.233	0.163
West	0.224	0.132	0.175	0.199	0.260	0.353
South	0.333	0.234	0.255	0.339	0.414	0.427
Total population (100,000)	1.047 [3.336]	0.517 [1.023]	0.975 [2.232]	0.967 [2.242]	1.374 [3.387]	1.407 [5.727]
Share of no internet access (%)	16.260 [7.207]	12.486 [4.693]	13.880 [5.477]	16.138 [6.373]	17.611 [7.462]	21.245 [8.083]
Active MDs per 100,000 population	126.802 [178.895]	111.594 [124.480]	148.240 [253.129]	126.080 [185.775]	136.900 [163.773]	111.262 [135.630]
Hospital beds per 1,000 population	2.871 [4.972]	3.059 [5.238]	2.673 [4.875]	2.582 [3.475]	2.872 [3.532]	3.165 [6.909]
Observations	3113	629	623	618	623	620

Source: 2020 CMS Medicare Advantage Landscape File, 2020 CDC SVI data, Area Health Resource File, and Rural-Urban Commuting Area Codes. Notes: This study used a county level unit of analysis to define a local market. The fifth quintile (“Very high”) is the quintile of markets in the U.S. that have the highest SVI percentile rankings (0.8 or higher), while the first quintile (“Very low”) has the lowest percentile rankings (lower than 0.2). Means are not weighted by market population. Standard deviations are shown for continuous variables in square brackets

The mean count of all MA plans per market was 16.3 (SD=10.7) and this average was lower in areas with the greatest social vulnerability (mean=13.5; SD=8.0). The number of *high-quality* MA plans was also lower in the most vulnerable markets. Specifically, these markets had only a mean of 0.2 high-quality MA plans (with 5 stars), 1.5 (SD=4.4) high-quality MA plans with 4.5 stars or higher, and six (SD=8.2) high-quality plans with 4 stars or higher.

**Figure 1:**
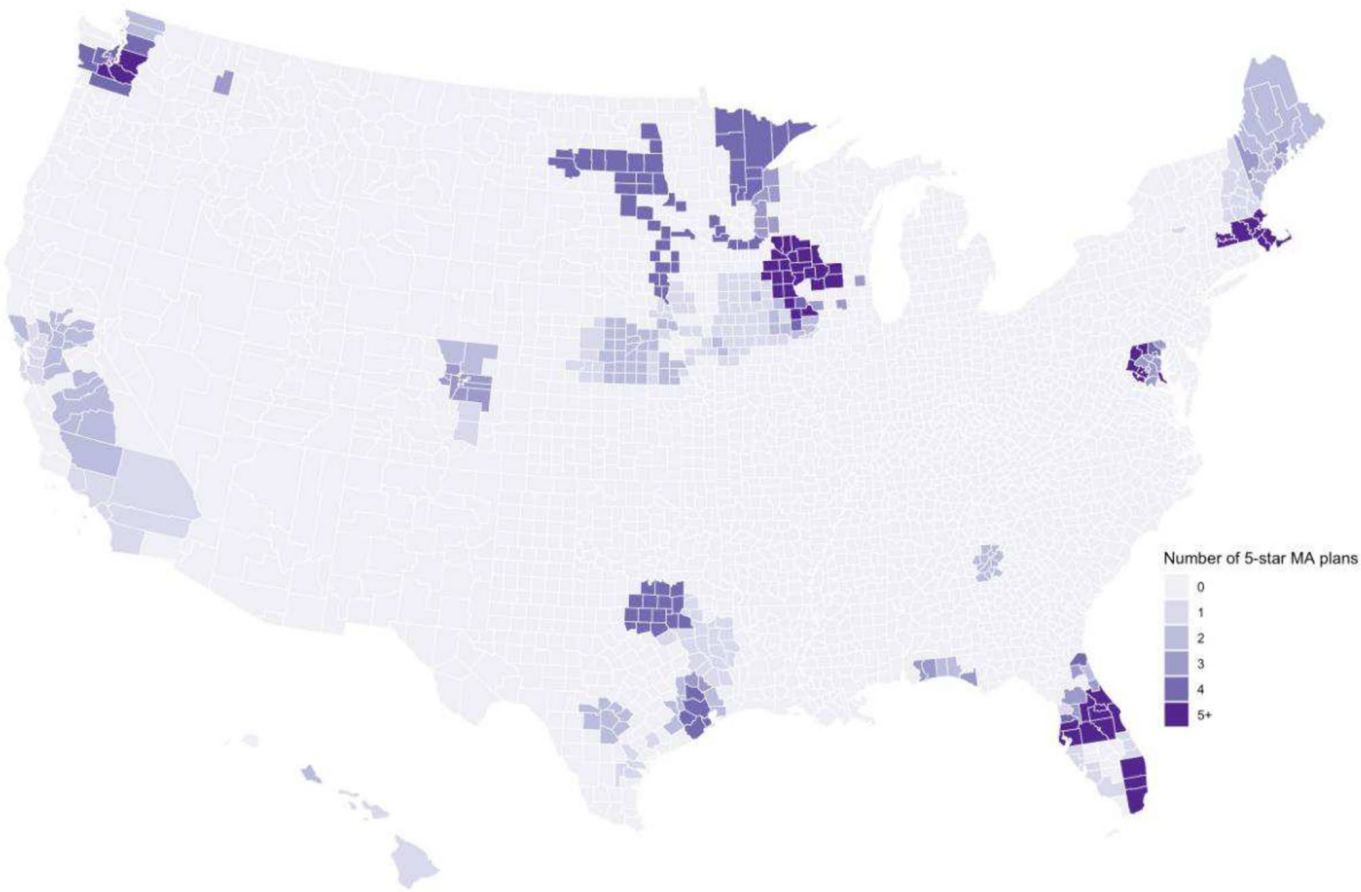
The number of 5-star MA plans at the market level, 2020. Source: 2020 CMS Medicare Advantage Landscape File.

Figure 1 shows the number of 5-star MA plans at the market level. In 2020, 86.5% of all markets did not have any 5-star plans. The share of markets without 5-star MA plans was particularly higher in the South (91.4%) than in the West (84.1%), Midwest (87.4%), and Northeast (76.1%).

In Table 2, we report findings from the multivariate regression analyses of the number of MA plans on SVI quintile groups (reference group is the lowest SVI quintile). We found evidence of significant and negative associations between market-level SVI scores and outcome measures. Compared to the least vulnerable markets, the most vulnerable markets had 1.5 fewer MA plans overall (*p*-value, 0.001). In addition, the most vulnerable markets had 0.68 fewer plans with 4.5 or higher stars (23.5% of the mean; *p*-value < 0.001), and 1.1 fewer 4+ star plans (12.1% of the mean; *p*-value < 0.001) than the least vulnerable markets.

**Table 2.** Association of Market-Level Social Vulnerability with Availability of High-Quality Medicare Advantage Plans

	Outcome measures			
	Number of 5-star MA plans	Number of 4.5+ star MA plans	Number of 4+ star MA plans	Number of all MA plans
SVI quintiles (reference=very low)				
Q2 (low)	0.017 [−0.143, 0.178]	−0.174 [−0.469, 0.121]	−0.240 [−0.685, 0.206]	−0.419 [−1.096, 0.258]
Q3 (moderate)	0.002 [−0.180, 0.184]	−0.262* [−0.560, 0.036]	−0.315 [−0.848, 0.219]	−0.463 [−1.180, 0.253]
Q4 (high)	−0.055 [−0.249, 0.140]	−0.303 [−0.668, 0.062]	−0.551* [−1.186, 0.083]	−0.547 [−1.509, 0.416]
Q5 (very high)	−0.159 [−0.361, −0.044]	−0.679*** [−1.132, −0.227]	−1.079*** [−1.862, −0.295]	−1.498** [−2.868, −0.128]
Outcome mean	0.385	2.894	8.947	16.290

*N*=3113. This study used a county-level unit of analysis to define a local market. 95% CIs are in square brackets. Robust standard errors are clustered at the state level. Each column represents separate regression result. Regressions control for rurality, % households without internet access, active MDs per 100k population, hospital beds per 100k population, and state fixed effects. Market-level outcome means are not weighted by population

*MA*, Medicare Advantage; *SVI*, Social Vulnerability Index

*, **, ***Significant at 0.1, 0.5, and 0.01

**Figure 2:**
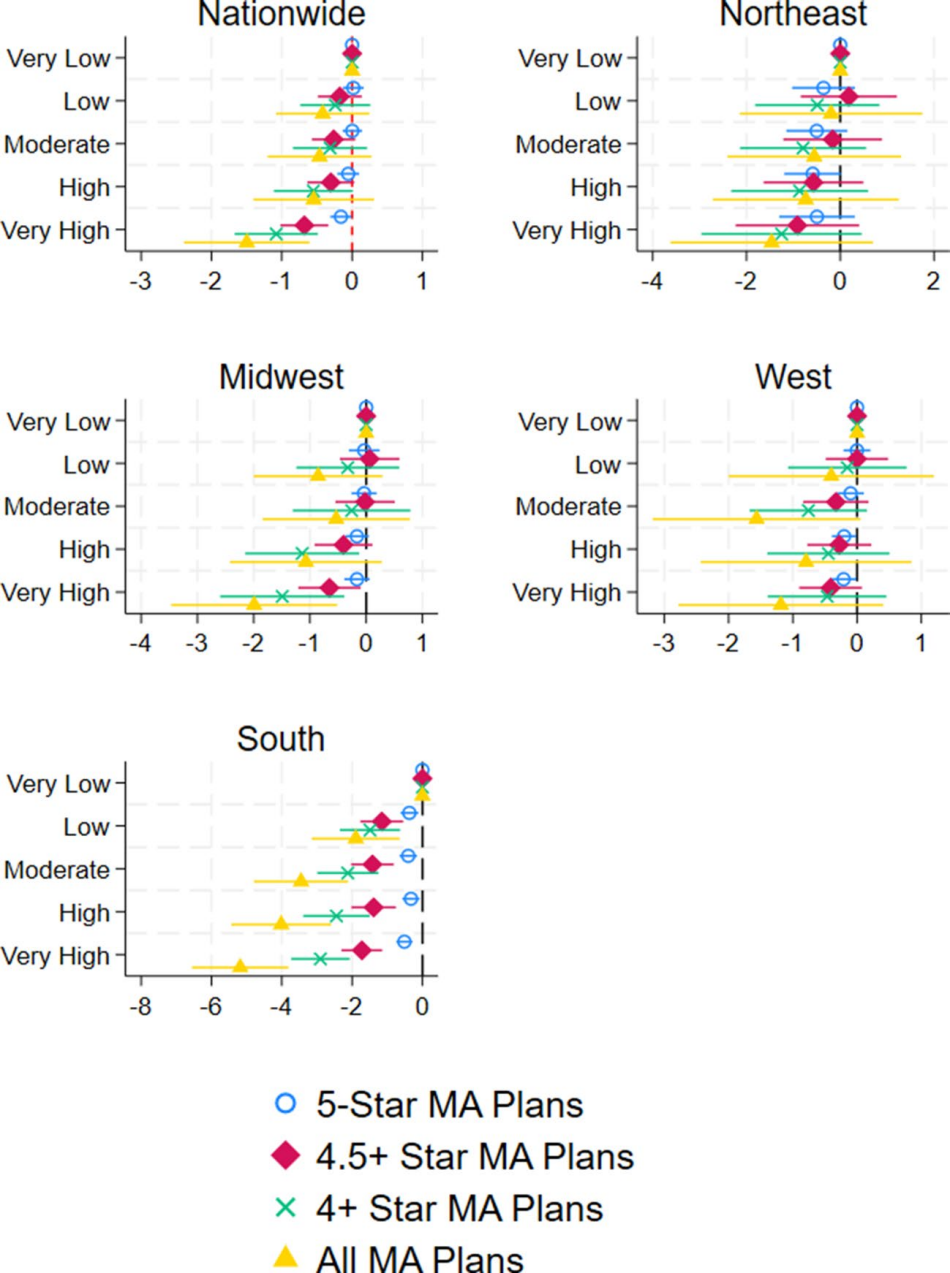
Association between market-level SVI percentile ranking and the availability of high-quality MA plans by census region. Graphs show regression coefficients and 95% confidence intervals taken from separate linear regressions controlling for covariates shown in Table 2. “Very low” SVI is the omitted group.

To explore disparities in access to high-quality MA plans, we performed stratified analyses by census region and rurality status. Figure 2 shows regression coefficients and 95% confidence intervals from separate regression analyses for each of the four Census regions. The market-level SVI’s negative associations with the number of high-quality MA plans (Table 2) were especially strong in the southern USA region. In the South, markets with the highest level of vulnerability had 0.5 fewer 5-star plans (*p*-value < 0.001), 1.7 fewer plans with 4.5+ stars (*p*-value < 0.001), and 2.9 fewer plans with 4+ stars (*p*-value < 0.001) compared to the least vulnerable markets.

**Figure 3:**
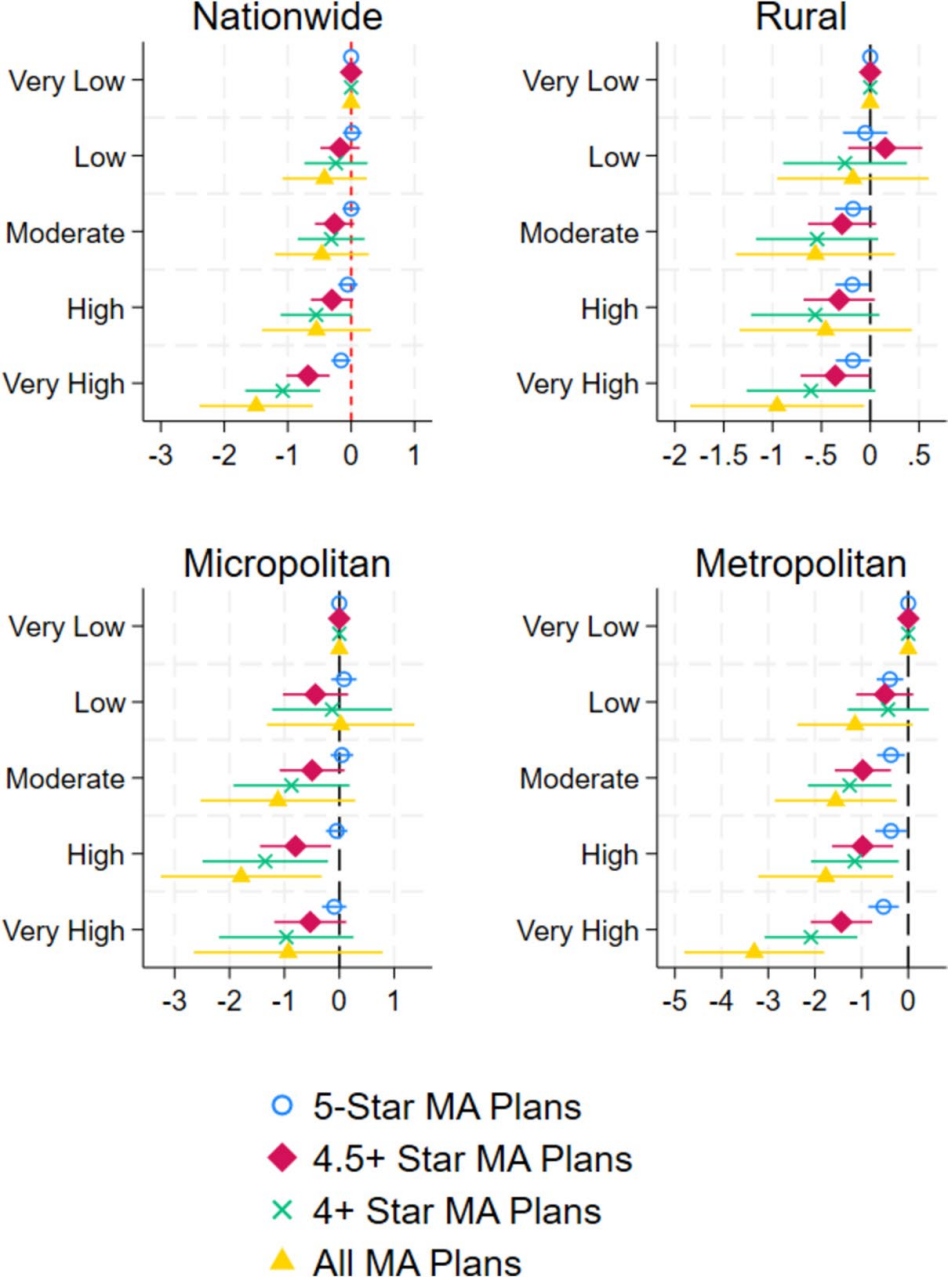
Association between market-level SVI percentile ranking and the availability of high-quality MA plans by rurality. Graphs show regression coefficients and 95% confidence intervals taken from separate linear regressions controlling for covariates shown in Table 1. “Very low” SVI is the omitted group.

Results from the subgroup analysis by rurality status (Fig. 3) indicate much stronger associations in metropolitan markets. In metropolitan areas, markets with the highest level of social vulnerability had 0.5 fewer 5-star MA plans (*p*-value, 0.001), 1.4 fewer 4.5+ star MA plans (*p*-value < 0.001), and 2.1 fewer 4+ star MA plans (*p*-value < 0.001) compared to the lowest SVI quintile markets. We did not find significant associations among micropolitan markets.

In Appendix Table 1, we ran regressions with a continuous SVI percentile ranking measure ranging from 0 (least vulnerable) to 1 (most vulnerable) for all markets (panel A), subgroups by census regions (panels B through E), and subgroups by rurality status (panels F through H). Overall, compared to the least vulnerable markets, the most vulnerable markets have 0.2 fewer 5-star MA plans (56.9% of the mean;* p*-value, 0.017). The strongest association of SVI percentile ranking with the number of 5-star MA plans occurred in the South. Also, the association was found to be strong among metropolitan markets.

Appendix Table 2 shows results using the four SVI subcategories as our regressor of interest. The strongest association with the availability of high-rated MA plans was found for the socioeconomic vulnerability index. The most socioeconomically vulnerable markets have significantly fewer 5-star MA plans (−0.2), 4.5+ star MA plans (−0.8), 4+ star MA plans (−1.1), and all MA plans (−1.5) compared to the least vulnerable market group. Notably, we did not find significant associations of the availability of high-rated MA plans with the household composition vulnerability index and the racial/ethnic minority vulnerability index.

## DISCUSSION

Although more than half of beneficiaries are enrolled in MA plans, disparities in access to high-quality MA plans at the market level based on social vulnerability have not been rigorously studied. We found that the number of high-quality MA plans is lower in markets with greater social vulnerability scores. In particular, this negative association was concentrated in the southern region, which has a greater proportion of Black/African Americans in its market-level populations. By 2027, CMS plans to implement a new Health Equity Index (HEI) score as part of the MA Star Ratings score in order to incentivize MA plans to focus more attention on socially vulnerable populations. These findings suggest that CMS can incorporate SVI measures to adjust HEI scores at the market level and improve access to high-quality MA plans in markets with greater social vulnerability scores.

Our overall results are consistent with prior studies reporting that high-rated MA plans are less likely to operate in markets with a higher share of racial/ethnic minorities.^13,14^ However, unlike these prior studies, we used a measure of SVI that incorporates market-level socioeconomic, household, and transportation factors in addition to race/ethnicity. We found that Medicare beneficiaries living in socially vulnerable markets, regardless of race/ethnicity, have worse access to high-rated MA plans than peers living in less vulnerable markets. Our results suggest that private insurers’ decisions to offer MA plans may not necessarily be directly related to racial/ethnic mix per se, but instead are closely related to a community’s degree of overall social vulnerability.

We also found that the number of high-quality MA plans and the number of all MA plans are significantly lower in the most socially vulnerable markets, indicating that private insurers are likely to avoid market entry of MA plans in local areas with greater unmet social needs. Also, these findings suggest the need to monitor the availability of high-quality MA plans in socially vulnerable markets to improve health equity. Finally, policy interventions or regulatory actions are needed to address disparities in beneficiary access to high-quality MA plans. Since the current star rating system assesses quality at the contract level, it may not provide adequately detailed information on MA plan quality at the local level. A new rating system assessing MA plan quality at the local level may better inform Medicare beneficiaries.^20^

### Limitations

This study has several data limitations. First, due to the cross-sectional study design, we could not draw causal inferences between area-level social vulnerability and the availability of high-quality MA plans. Since we could not account for all factors correlated with the SVI measure and the number of MA plans in each market, unobserved factors may bias our estimates. Second, our data do not represent all MA plans as we excluded those available only to subpopulations of beneficiaries, such as SNPs. Third, our data may not reflect current trends in the MA market because 2020 SVI scores were the most recent data available. As MA enrollment has continued to grow even after COVID-19 lockdowns subsided, our results may change as more recent data become available.

### CONCLUSION

Beginning in 2027, CMS plans to implement a new HEI score to reward MA contracts and thereby encourage private insurers to improve access to care and plan availability for disadvantaged beneficiaries.^21^ Our findings suggest the need for policymakers and CMS to monitor access to higher-quality MA plans for lower-income and disadvantaged beneficiaries on an annual basis. While such efforts are valuable at the state level, it is also important to monitor the availability of high-quality MA plans at the local market level. Finally, this study demonstrates the feasibility of using the SVI score as an indicator of social vulnerability to monitor disparities in access to high-quality MA plans.

## Supplementary Information

Below is the link to the electronic supplementary material.

Supplementary file1 (DOCX 22.2 KB)
