# Supplementary material for: Association of Social Vulnerability and Access to Higher Quality Medicare Advantage Plans
Source: J Gen Intern Med. 2024 Dec 20;40(8):1869–76. doi: 10.1007/s11606-024-09252-1 (PMC12119444; doi:10.1007/s11606-024-09252-1)
Supplement: Supplementary file 1 — Supplementary file1 (DOCX 22.2 KB) [file 11606_2024_9252_MOESM1_ESM.docx]

Appendix Table 1. Association of county-level social vulnerability with availability of high-quality Medicare Advantage plans: With a continuous SVI percentile ranking measure

|  | Outcome measures | | | |
| --- | --- | --- | --- | --- |
|  | # of 5-star plans | # of 4.5+ star plans | # of 4+ star plans | # of all MA plans |
| *Panel A. All counties (N=3,113)* | | | | |
| SVI percentile rankings (0 to 1) | -0.219  [-0.444, 0.005] | -0.743^***^  [-1.209, -0.276] | -1.147^**^  [-2.047, -0.246] | -1.329^*^  [-2.911, 0.253] |
| Outcome mean | 0.385 | 2.894 | 8.947 | 16.290 |
| *Panel B. Northeastern counties (N=394)* | | | | |
| SVI percentile rankings (0 to 1) | -0.315  [-0.814, 0.183] | -1.341  [-3.086, 0.405] | -0.884  [-3.902, 2.135] | -0.576  [-3.788, 2.635] |
| Outcome mean | 0.721 | 5.609 | 12.292 | 18.224 |
| *Panel C. Midwestern (N=983)* | | | | |
| SVI percentile rankings (0 to 1) | -0.290  [-0.664, 0.083] | -1.117^**^  [-1.927, -0.306] | -2.255^**^  [-3.886, -0.624] | -2.624^**^  [-4.597, -0.651] |
| Outcome mean | 0.338 | 2.808 | 9.824 | 16.848 |
| *Panel D. Western counties (N=696)* | | | | |
| SVI percentile rankings (0 to 1) | -0.341^*^  [-0.744, 0.063] | -0.502  [-1.426, 0.421] | -0.324  [-1.759, 1.111] | -0.608  [-2.191, 0.974] |
| Outcome mean | 0.332 | 1.445 | 4.534 | 12.130 |
| *Panel E. Southern counties (N=1,040)* | | | | |
| SVI percentile rankings (0 to 1) | -0.605^**^  [-1.122, -0.088] | -1.887^***^  [-3.051, -0.724] | -3.501^***^  [-4.851, -2.150] | -6.688^***^  [-9.421, -3.954] |
| Outcome mean | 0.339 | 2.917 | 9.804 | 17.737 |
| *Panel F. Rural counties (N=1,276)* | | | | |
| SVI percentile rankings (0 to 1) | -0.268^**^  [-0.517, -0.019] | -0.729^***^  [-1.183, -0.275] | -1.212^***^  [-2.123, -0.301] | -1.475^**^  [-2.770, -0.180] |
| Outcome mean | 0.257 | 1.507 | 5.885 | 11.620 |
| *Panel G. Micropolitan counties (N=659)* | | | | |
| SVI percentile rankings (0 to 1) | -0.123  [-0.474, 0.228] | -0.553  [-1.443, 0.338] | -1.208  [-2.943, 0.526] | -1.693  [-4.467, 1.081] |
| Outcome mean | 0.191 | 2.495 | 8.429 | 15.011 |
| *Panel H. Metropolitan counties (N=1,178)* | | | | |
| SVI percentile rankings (0 to 1) | -0.315  [-0.796, 0.166] | -1.155^**^  [-2.044, -0.267] | -1.986^**^  [-3.580, -0.393] | -2.603^**^  [-4.955, -0.252] |
| Outcome mean | 0.633 | 4.621 | 12.553 | 21.909 |

Notes: Unit of analysis is county. 95% CIs are in square brackets. Standard errors are clustered at the state level. Each cell represents separate regression result. Regressions control for rurality, % households without internet access, active MDs per 100k population, hospital beds per 100k population, and state fixed effects. County-level outcome means are not weighted by population.

*, **, ***: significant at 0.1, 0.05, and 0.01.

Appendix Table 2. Association of county-level social vulnerability with availability of high-quality Medicare Advantage plans: With subcategories of SVI

|  | Outcome measures | | | |
| --- | --- | --- | --- | --- |
|  | # of 5-star plans | # of 4.5+ star plans | # of 4+ star plans | # of all MA plans |
| *Panel A. Socioeconomic vulnerability quintiles (reference=very low)* | | | | |
| Q2 (Low) | -0.140*  [-0.308, 0.027] | -0.281  [-0.793, 0.231] | -0.450*  [-1.119, 0.218] | -0.401  [-1.146, 0.344] |
| Q3 (Moderate) | -0.157  [-0.361, 0.048] | -0.623**  [-1.177, -0.070] | -0.819**  [-1.522, -0.117] | -0.695  [-1.680, 0.289] |
| Q4 (High) | -0.153**  [-0.297, -0.009] | -0.597**  [-1.053, -0.141] | -0.976**  [-1.764, -0.187] | -1.057**  [-2.078, -0.036] |
| Q5 (Very high) | -0.225***  [-0.380, -0.071] | -0.781***  [-1.256, -0.306 | -1.131**  [-1.991, -0.271] | -1.460**  [-2.841, -0.080] |
| *Panel B. Household composition vulnerability quintiles (reference=very low)* | | | | |
| Q2 (Low) | -0.022  [-0.168, 0.123] | 0.083  [-0.203, 0.368] | 0.266  [-0.246, 0.778] | 0.487  [-0.221, 1.195] |
| Q3 (Moderate) | -0.028  [-0.218, 0.161] | -0.058  [-0.493, 0.375] | 0.019  [-0.648, 0.685] | 0.572  [-0.369, 1.513] |
| Q4 (High) | -0.035  [-0.241, 0.172] | 0.052  [-0.361, 0.464] | 0.166  [-0.496, 0.828] | 0.557  [-0.379, 1.492] |
| Q5 (Very high) | -0.099  [-0.293, 0.096] | -0.245  [-0.626, 0.136] | -0.209  [-0.900, 0.482] | -0.102  [-1.108, 0.905] |
| *Panel C. Racial/Ethnic vulnerability quintiles (reference=very low)* | | | | |
| Q2 (Low) | -0.050  [-0.171, 0.071] | -0.096  [-0.478, 0.285] | -0.027  [-0.478, 0.414] | -0.317  [-0.957, 0.323] |
| Q3 (Moderate) | -0.283***  [-0.442, -0.122] | -0.135  [-0.583, 0.313] | 0.066  [-0.681, 0.812] | 0.474  [-0.469, 1.417] |
| Q4 (High) | -0.167*  [-0.335, 0.002] | -0.166  [-0.822, 0.491] | -0.141  [-1.116, 0.834] | 0.640  [-0.694, 1.975] |
| Q5 (Very high) | -0.012  [-0.442, 0.418] | -0.237  [-1.012, 0.538] | -0.422  [-1.553, 0.709] | 0.770  [-1.079, 2.619] |
| *Panel D. Housing & Transportation vulnerability quintiles (reference=very low)* | | | | |
| Q2 (Low) | 0.067  [-0.066, 0.199] | 0.047  [-0.219, 0.313] | 0.462*  [-0.035, 0.960] | 0.277  [-0.464, 1.018] |
| Q3 (Moderate) | 0.048  [-0.106, 0.201] | 0.290*  [-0.055, 0.635] | 0.257  [-0.294, 0.807] | -0.051  [-0.936, 0.835] |
| Q4 (High) | 0.027  [-0.146, 0.200] | 0.016  [-0.339, 0.370] | 0.069  [-0.487, 0.624] | -0.295  [-1.185, 0.595] |
| Q5 (Very high) | -0.066  [-0.257, 0.125] | -0.484*  [-0.972, 0.005] | -0.770**  [-1.518, -0.021] | -1.257**  [-2.441, -0.074] |
| Outcome mean | 0.385 | 2.894 | 8.947 | 16.290 |

Notes: N=3,113.

Unit of analysis is county. 95% CIs are in square brackets. Robust standard errors are clustered at the state level. Each column represents separate regression result. Regressions control for rurality, % households without internet access, active MDs per 100k population, hospital beds per 100k population, and state fixed effects. County-level outcome means are not weighted by population.

*, **, *** represent significant at 0.1, 0.5, and 0.01.
